# Laser frequency noise characterization using high-finesse plano–concave optical microresonators

**DOI:** 10.1364/OL.510516

**Published:** 2024-01-29

**Authors:** David Martin-Sanchez, Edward Z. Zhang, Jake Paterson, James A. Guggenheim, Zhixin Liu, Paul C. Beard

**Affiliations:** 1 Department of Medical Physics and Biomedical Engineering, University College London, UK; 2 Instituto de Microelectrónica de Sevilla (IMSE-CNM), CSIC-Universidad de Sevilla, Spain; 3 Wellcome/EPSRC Centre for Interventional and Surgical Sciences, University College London, UK; 4 Department of Electronic and Electrical Engineering, University College London, UK; 5 Institute of Cardiovascular Sciences, College of Medical and Dental Sciences, University of Birmingham, UK; 6 School of Engineering, College of Engineering and Physical Sciences, University of Birmingham, UK

## Abstract

Characterizing laser frequency noise is essential for applications including optical sensing and coherent optical communications. Accurate measurement of ultra-narrow linewidth lasers over a wide frequency range using existing methods is still challenging. Here we present a method for characterizing the frequency noise of lasers using a high-finesse plano–concave optical microresonator (PCMR) acting as a frequency discriminator. To enable noise measurements at a wide range of laser frequencies, an array of PCMRs was produced with slight variations of thickness resulting in a series of discriminators operating at a series of periodical frequencies. This method enables measuring the frequency noise over a wide linewidth range (15 Hz to <100 MHz) over the 1440–1630 nm wavelength range. To assess the performance of the method, four different lasers were characterized, and the results were compared to the estimations of a commercial frequency noise analyzer.

## Introduction.

The output of a single-wavelength laser source is not perfectly monochromatic but has a finite linewidth. This is manifested as frequency noise, which is typically measured as the power spectral density (*PSD_ν_*). Frequency noise is a key performance indicator of a laser as it can be used to describe its stability and to analytically derive a laser linewidth [1], which is fundamental to determining the resolution in frequency metrology [2], sensitivity of an optical coherent sensing system [3], and the spectrum efficiency in coherent optical communications [4].

Several techniques have been developed to characterize a laser linewidth. One well-established method involves heterodyning the laser output with a reference beam provided by an uncorrelated laser. This results in an RF beat signal whose frequency fluctuations are a combination of the individual frequency fluctuations of the two laser sources [5]. The uncorrelated reference beam can also be provided by the same laser source by using a delay line longer than the coherence length of the laser [6]. However, this approach requires impractically long delay lines for characterizing narrow linewidth lasers with sub-kHz linewidths, for example, those used for high-speed optical coherent communications [7,8] and gravitational wave detection [9].

The frequency noise spectrum can be determined by the use of frequency discriminators to transform the frequency fluctuations of the laser source into real-time variations of the optical intensity [5]. Mach–Zehnder interferometers [10] and Fabry–Perot (FP) interferometers [11] have been successfully used for this purpose. However, frequency discriminators present several limitations. One is the limitation on the dynamic range, which restricts the operation to a narrow frequency noise spectrum. In addition, discriminators are designed at a specific optical frequency, limiting their application to tuneable lasers. Furthermore, in the case of FP interferometers, it is challenging to realize sufficiently high finesse in order to characterize narrow linewidth lasers (maximum finesse reported was 70 by Ref. [12]).

In this Letter, a compact high-resolution tuneable frequency discriminator is proposed for measuring laser frequency noise that can overcome the limitations of existing methods. It is based on a dense array of 5000 plano–concave optical microresonators (PCMRs) [13]. The use of the plano–concave geometry, high mirror reflectivities, and a low absorbing optical material enables high finesse to be achieved allowing the measurement of laser linewidths as low as 15 Hz. The system also allows linewidths up to 100 MHz to be measured. By designing the PCMRs to have a low FSR and exploiting the random manufacturing variations in optical thickness between different PCMRs in the array, frequency noise measurements can be made over a wide laser wavelength range, covering the S-, C-, and L-bands (1440–1630 nm). To evaluate the method, we used the discriminator to characterize four lasers with significantly different nominal linewidths and found good agreement with measurements made using a commercial frequency noise analyzer.

## Methods.

**Fig. 1.**
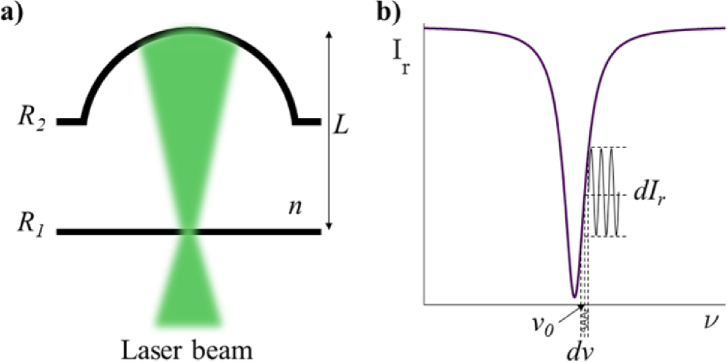
(a) Illustration of a PCMR illuminated by a focused Gaussian laser beam, and (b) cavity transfer function (CTF); reflected optical intensity *I_r_* of the PCMR as a function of the laser frequency *ν* showing a single cavity resonance. At the laser frequency *ν*_0_, frequency fluctuations *dν* are converted to an optical intensity modulation *dI_r_*.

The system comprises an array of plano–concave microresonators (PCMRs), each of which consists of a planar mirror and a spherical mirror of reflectivities *R*_1_ and *R*_2_, respectively separated by a spacer of refractive index *n* and thickness *L* (Fig. 1(a)). The reflected optical intensity, *I_r_*, of the PCMR when illuminated by a focused Gaussian beam such that the curvature of the spherical mirror matches that of the beam wavefront follows the Airy Function [14,15]:

(1) $$I_{r}=\frac{{\left(\sqrt{R_{1}}-\sqrt{R_{2}}\right)}^{2}+4\sqrt{R_{1}\cdot R_{2}}\cdot sin^{2}(\pi \nu /FSR)}{{\left(1-\sqrt{R_{1}\cdot R_{2}}\right)}^{2}+4\sqrt{R_{1}\cdot R_{2}}\cdot sin^{2}(\pi \nu /FSR)},$$

where *ν* is the optical frequency, and FSR is the free spectral range of the PCMR defined at a normal incidence as *FSR = c/2nL*. At frequencies corresponding to integer multiples of the FSR, the light reflected from the two mirrors interferes and resonates, producing a series of equally spaced minima in the reflected optical intensity. One such reflectance minimum is illustrated in Fig. 1(b) and is referred to as the cavity transfer function (CTF).

As Fig. 1(b) illustrates, when the central frequency of the laser *ν*_0_ lies on the edge of the CTF, frequency fluctuations *dν* in the laser output are converted to corresponding intensity variations *dI_r_*. In this way the PCMR serves as a frequency discriminator that can be used to measure the laser frequency noise. For a given PCMR CTF, the sensitivity and linearity are defined by *ν*_0_. When *ν*_0_ is aligned to the maximum slope of the CTF, the frequency sensitivity and linearity are greatest.

The PCMR is insensitive to frequency fluctuations at laser frequencies where the CTF is flat (i.e., between cavity resonances), precluding the measurement of frequency noise. To address this limitation, an array of multiple PCMRs was used. Each PCMR was nominally identical except for the thickness L which exhibited slight variations due to manufacturing tolerances. The periodic cavity resonances for each PCMR therefore occur at slightly different frequencies and thus “fill” in the gaps between the resonances of any single PCMR. Given a sufficiently high density of resonances, a measurement of frequency noise can be made at any laser wavelength by selecting an appropriate PCMR within the array.

**Fig. 2.**
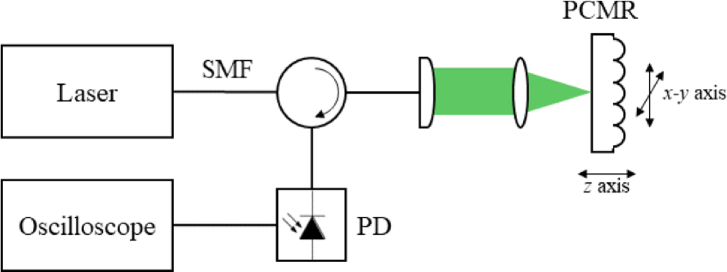
Experimental setup used to characterize the frequency noise of laser sources using a PCMR array.

The practical implementation of this approach is shown in Fig. (2). The PCMR array comprises 5000 fused silica plano–convex structures, each of nominal thickness *L *= 1.2 mm, with variations within ±3% due to the manufacturing process, and coated with high reflectivity (>90%) mirrors in the 1150–1650 nm range (at 1550 nm *R*_1 _= 98.6% and *R*_2 _= 98.4%).

A system similar to the one described in Ref. [16] was initially employed to characterize the array. To address an individual PCMR within the array, a translation stage was then used to position the focused laser beam at the center of the PCMR. The focused beam was formed by coupling the laser output into a single mode fiber (SMF) with MFD = 10.4 ± 0.5 µm. The output of the SMF was then focused onto the first mirror of the PCMR using a collimator and an objective lens (F240APC-1550 and pair of LA1134-C, respectively, Thorlabs Inc.) in a 4f configuration selected to produce a focal beam waist of 38 µm that matched the curvature of the spherical mirror surface. The beam reflected from the PCMR is coupled back onto an AC and a DC-coupled InGaAs photodiode (PD) with a 900–1700 nm spectral range (G9801-22, Hamamatsu Photonics) via an optical circulator. The AC voltage signal was acquired using a 50 Ω coupled oscilloscope (TDS5034B, Tektronix Inc.) with a 350 MHz analog bandwidth and a sampling rate of 5 GHz. The recorded waveforms were then acquired by a PC.

In order to make a quantitative measurement of the frequency noise, knowledge of the CTF is required. The CTF was acquired by sweeping the wavelength of a tuneable external cavity CW laser (Tunics T100S-HP/SCL, Yenista Optics) over the range 1440–1630 nm while recording the DC-coupled output of the photodiode, *V_DC_*, which was then divided by *P*(*λ*), a scaling factor representing the laser optical power variation with the wavelength normalized to the maximum power output [16].

With the laser frequency aligned to the edge of a cavity resonance as shown in Fig. 1(b), the time-varying optical intensity modulation due to the laser frequency noise *dν*(*t*) was acquired by recording the AC-coupled photodiode voltage signal *V_AC_*(*t*). Then *dν*(*t*) was estimated as follows:

(2) $$d\nu (t)=\frac{2\cdot V_{AC}(t)}{{dI}_{r}^{bias}/d\lambda \cdot P_{0}\cdot dV/dP\cdot G}\cdot c/\lambda_{0}^{2},$$

where *λ*_0_ is the central wavelength of the laser source, *G *= 9 accounts for the gain difference between the DC and AC outputs of the PD, the factor of 2 accounts for the 50 Ω termination of the AC-coupled PD output, *dI_r_^bias^/dλ* is the normalized gradient of the CTF at *λ_0_* measured in nm^−1^, and *P*_0_*·dV/dP* converts *dI_r_^bias^/dλ* to the voltage gradient (V/nm), where *P*_0_ denotes the laser output power measured at the output of the SMF, and *dV/dP* is a conversion factor obtained by measuring *I_r_* at a wavelength where the CTF is flat. The power spectral density of the frequency noise is then given by the Fourier transform of *dν*(*t*):

(3) $$PSD_{\nu }(f)=F(d\nu (t)).$$

Assuming the spectral content of the laser beam has a Lorentzian line shape, its full width at half maximum (FWHM) can be approximated to provide a measure of the laser linewidth *Δν* [17] as follows:

(4) $$\Delta v=2\pi \;PSD_{v},$$

where *PSD_ν_* is the frequency noise in Hz^2^/Hz.

The practical operation of the system requires locating a cavity resonance that aligns with the laser wavelength. When using a fixed wavelength laser, the array is mechanically *x*−*y* scanned whilst monitoring the power from each PCMR in turn. The PCMR with a reflected optical power that corresponds approximately to the maximum CTF slope is then selected and used to measure *PSD_ν_*(*f*).

## Results.

The PCMR characteristics were determined by measuring the CTF using the tuneable external cavity CW laser. The combination of high mirror reflectivities, the plano–concave structure which minimizes beam walk-off and the low optical attenuation of fused silica conspire to provide a highly resonant cavity with a maximum Q-factor of 800,000, FWHM = 2 pm and finesse of 350. The measured FSR was 700 pm. To determine the upper limit of linear detection, a number of CTFs were measured. At the point of maximum slope on the CTF, it was observed that the CTF is linear to within +/−5% over a frequency range Δf–FWHM/8. Hence, for the measured FWHM of 2 pm, the maximum laser linewidth that can be measured at 1550 nm is *Δf = *100 MHz.

As discussed above, the existence of a PCMR cavity resonance that aligns with the laser wavelength is required to make a measurement. Based on the 5000 PCMRs in the array and the number of resonances (200) over the 1440–1630 nm wavelength range, there are over one million resonances that can be assumed to be randomly distributed, corresponding to a mean fringe spacing of 0.2 pm. Given this and the 1.25 pm linear wavelength range of an individual PCMR, it can reasonably be assumed there will be sufficient overlapping resonances to allow a measurement of the frequency noise at any wavelength between 1440 and 1630 nm.

To demonstrate the system, four lasers of different linewidths (according to their datasheets) were evaluated and compared to a commercially available frequency noise analyzer (OE4000, OEwaves Inc.). This instrument has the ability to measure ultra-low frequency noise (0.2 Hz/√Hz @ 1 MHz) of CW laser sources in the 1530–1565 nm range. It is based on a self-homodyne technique [18] that employs at least two interferometers with different delay-line designs. For the comparison, the instrument was configured to measure the frequency noise from 10 kHz to 10 MHz in 1-decade steps, averaging 100 times.

**Fig. 3.**
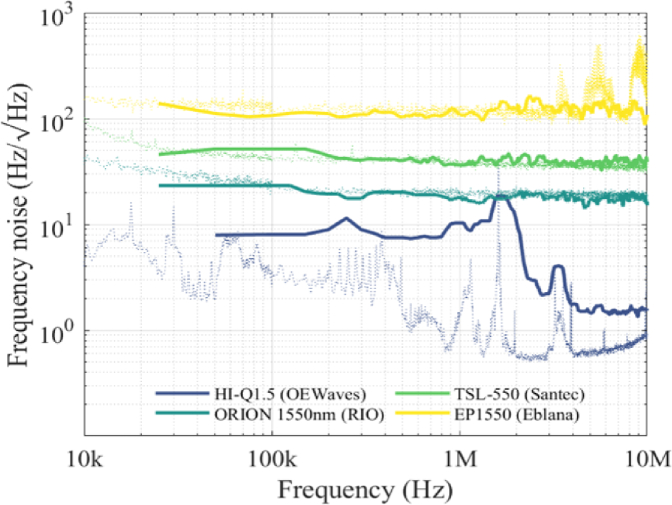
Power spectral density of the frequency noise measured for four different laser sources using the PCMR array frequency discriminator (solid lines) and the commercial frequency noise analyzer (dotted lines).

The four lasers that were evaluated were: (1) HI-Q 1.5 MICRON laser module from OEWaves Inc. with an ultra-narrow linewidth of 40 Hz, (2) ORION 1550 nm laser module from Redfern Integrated Optics Inc. with a 3 kHz linewidth, (3) TSL-550 compact laser from Santec Corporation with a 20 kHz linewidth, and (4) EP1550 laser module from Eblana Photonics Ltd. with a broad linewidth (60 kHz). RIN was <−150 dB/Hz in all cases. The *P*_0_ of each laser was 14.3, 14, 15, and 11.5 mW, respectively. The measured *dI_r_^bias^/dλ* of the CTF at the wavelength of each laser source was 270, 250, 280, and 300 nm^−1^, respectively. The conversion factor *dV/dP* in each case was 0.22, 0.24, 0.22, and 0.21 V/mW, respectively. For each laser, 10 different recordings of the AC-coupled photodiode voltage signal *V_AC_*(*t*) were captured using the system shown in Fig. (2) and converted to *PSD_ν_* using Eqs. (2) and (3). Then, the frequency noise spectra were averaged. The square root of the *PSD_ν_* is shown in Fig. (3) in the range from 10 kHz to 100 MHz after applying a median filter of the 10^th^ order.

Overall, the measured frequency noise spectra agree with the expected behavior for each laser: those with a wider linewidth exhibit higher frequency noise. The TSL-550, ORION 1550 nm, and EP1550 lasers display a typical white frequency noise spectrum across most of the frequency range, whereas at lower frequencies the noise increases, mostly due to flicker noise following a 1/f trend. The HI-Q 1.5 MICRON displays a low frequency noise of 1.5 Hz/√Hz at 10 MHz, which corresponds to a linewidth of 15 Hz. The ability to measure such a narrow linewidth is a consequence of the high finesse of the PCMRs. To further quantify the comparison the laser linewidth was calculated for each laser using Eq. (4), neglecting the contribution of RIN. The values obtained at 0.1 MHz can be seen in Table 1.

**Table 1.** Laser Linewidth at 0.1 MHz for Several Lasers Calculated with the Proposed Method and Compared to the Values Obtained from a Commercial Linewidth Analyzer

Laser	PCMR Array	Linewidth Analyzer
HI-Q1.5	0.4 kHz	0.1 kHz
ORION 1550 nm	3.3 kHz	4.2 kHz
TSL-550	16.7 kHz	11.1 kHz
EP1550	82.6 kHz	111.3 kHz

As illustrated in Fig. (3) and Table 1, good agreement is observed between the PMCR measurements and those obtained using the commercial frequency noise analyzer, except for the laser with the lowest frequency noise (HI-Q 1.5 MICRON laser module from OEWaves). Although the shape of its frequency noise spectrum is broadly in agreement, the absolute noise values obtained using the PCMR array are higher: at 0.1 MHz a difference of approximately 5 Hz/√Hz was measured, a difference that is also reflected in the linewidth measurement in Table 1 and is attributed to the shot noise from the PD. To quantify the effect of the shot noise, it was measured at a wavelength where the PCMR is not in resonance and thus is insensitive to the laser frequency noise. On average, the measured rms-value of the shot noise was <1 mV. This means that the shot noise accounts for approximately 3 Hz/√Hz of the frequency noise at 0.1 MHz. Hence, the results accurately represent the behavior of the lasers when their frequency noise exceeds that level by an appreciable margin.

These results demonstrate the feasibility of the PCMR method to characterize a wide range of laser frequency noise over a wide range of frequencies.

## Conclusions.

A method based on a frequency discriminator created by an array of PCMRs for characterizing the frequency noise of laser sources has been presented. Finesse 5× higher than previously reported [12] was achieved using high mirror reflectivities, a plano–concave geometry, and low optical attenuation inside the cavity. The method enables the laser frequency noise spectrum to be obtained from a single measurement from which linewidths as low as 15 Hz and up to 100 MHz over the 1440–1630 nm wavelength can be estimated. This makes the system suitable for characterizing single-frequency laser sources emitting in the S-, C-, and L-bands.

As well as a high sensitivity, a large dynamic range, and a wide operating wavelength range, the concept offers a design flexibility unavailable with conventional linewidth analyzers. The characteristics of the PCMR CTF can readily be adjusted by varying the mirror reflectivities and cavity spacing to modify the finesse and therefore the frequency noise measurement range: higher finesse will increase the frequency sensitivity enabling smaller linewidths (sub-Hz) to be measured, and reducing the finesse serves to increase the maximum measurable linewidth. Indeed, an array could be populated with PCMRs of different finesse that span a multitude of frequency noise measurement ranges. Although the current system was designed for use with CW lasers operating at optical telecom wavelengths, other wavelength ranges can be accommodated by an appropriate choice of the PCMR mirror design. The method could also potentially be applied to pulsed lasers. Moreover, a practical engineered system could be compact and of relatively low cost, allowing high-speed measurements.

In summary, it is considered that the concept offers a practical high-performance alternative to the current delay-line based frequency noise analyzer for characterizing narrow linewidth laser sources.

## Data Availability

Data underlying the results presented in this paper are available in Ref. [19].

## References

[1] Di Domenico G. Schilt S. Thomann P., Appl. Opt. 49, 4801 (2010). 10.1364/AO.49.004801 20820223

[2] Lamperti M. Gotti R. Gatti D. et al., Commun. Phys. 3, 175 (2020). 10.1038/s42005-020-00441-y

[3] Duan L. Zhang H. Shi W. et al., Sensors 18, 3245 (2018). 10.3390/s18103245 30261692 PMC6210321

[4] Dhawan D. Gupta N., Opt. Photonics J. 07, 92 (2017). 10.4236/opj.2017.75009

[5] Turner L. D. Weber K. P. Hawthorn C. J. et al., Opt. Commun. 201, 391 (2002). 10.1016/S0030-4018(01)01689-3

[6] Okoshi T. Kikuchi K. Nakayama A., Electron. Lett. 16, 630 (1980). 10.1049/el:19800437

[7] Huang D. Tran M. A. Guo J. et al., Optica 6, 745 (2019). 10.1364/OPTICA.6.000745

[8] Guan H. Novack A. Galfsky T. et al., Opt. Express 26, 7920 (2018). 10.1364/OE.26.007920 29715766

[9] Abbott B. P. Abbott R. Abbott T. D. et al., Phys. Rev. Lett. 116, 061102 (2016). 10.1103/PhysRevLett.116.061102 26918975

[10] Slavik R. Liao Y. Austin E. et al., in 21st International Conference on Optical Fiber Sensors, Vol. 7753 (2011), p. 775338.

[11] Kourogi M. Ohtsu M., Opt. Commun. 81, 204 (1991). 10.1016/0030-4018(91)90639-U

[12] Tremblay P., IEEE Trans. Instrum. Meas. 40, 204 (1991). 10.1109/TIM.1990.1032916

[13] Guggenheim J. A. Li J. Allen T. J. et al., Nat. Photonics 11, 714 (2017). 10.1038/s41566-017-0027-x

[14] Vaughan J. M., *The Fabry–Perot Interferometer* , 1st ed (Routledge, 2017).

[15] Martin-Sanchez D. Li J. Zhang E. Z. et al., Opt. Express 31, 16523 (2023). 10.1364/OE.484212 37157729 PMC11146662

[16] Zhang E. Beard P., IEEE Trans. Ultrason., Ferroelect., Freq. Contr. 53, 1330 (2006). 10.1109/TUFFC.2006.1665081 16889340

[17] Zhang Z. Yariv A., IEEE J. Quantum Electron. 56, 1 (2020). 10.1109/JQE.2020.2980011

[18] Ludvigsen H. Tossavainen M. Kaivola M., Opt. Commun. 155, 180 (1998). 10.1016/S0030-4018(98)00355-1

[19] Martin-Sanchez D. Liu Z. Zhang E. Z. et al., “Time-resolved frequency noise measurement using tunable frequency discriminator,” GitHub (2022), https://github.com/marsandav/LinewidthMeasurement.

